# Effects of Head Position on Perception of Gravity in Vestibular Neuritis and Lateral Medullary Infarction

**DOI:** 10.3389/fneur.2018.00060

**Published:** 2018-02-12

**Authors:** Sung-Hee Kim, Ji-Soo Kim

**Affiliations:** ^1^Department of Neurology, Kyungpook National University School of Medicine, Kyungpook National University Chilgok Hospital, Daegu, South Korea; ^2^Department of Neurology, Seoul National University College of Medicine, Seoul National University Bundang Hospital, Seoul, South Korea

**Keywords:** vertigo, subjective visual vertical, otolith organ, gravity, vestibular disease

## Abstract

**Objective:**

Internal representation of gravity can be quantified by measuring the subjective visual vertical (SVV). Modulation of verticality perception during head tilts may be perturbed in vestibular disorders causing SVV tilts in the upright head position. This study aimed to determine the influence of head tilts on the estimation of SVV in acute vestibular disorders.

**Methods:**

We measured the SVV in 37 patients with acute vestibular symptoms due to unilateral vestibular neuritis (VN) (*n* = 28) and lateral medullary infarction (LMI) (*n* = 9). Measurements of the SVV were performed under head upright, head tilt 30° and 60° in each direction. Seventeen normal subjects served as the control.

**Results:**

In controls, head tilt of 30° produced a contraversive shift of the SVV (the E-effect), and head tilt of 60° generated an ipsiversive shift (the A-effect). Patients with VN showed only the A-effect irrespective of the direction and amplitude of head tilt. Patients with LMI could estimate earth verticality accurately during head tilts. Patients with VN during the recovery phase showed the patterns of SVV modulation similar to those observed in the controls either with head upright or tilted.

**Conclusion:**

Given the absence of the E-effect in acute VN, the peripheral otolithic inputs appear to be essential in the perception of earth vertical during small static head tilts.

## Introduction

Internal representation of gravity can be quantified in a standard way by setting a luminous rod along the perceived vertical in darkness, the subjective visual vertical (SVV). While visual, vestibular, and somatosensory information all contributes to internal estimation of the verticality (1), the otolithic input is most important. In normal subjects, the SVV in the head upright position is aligned with the gravitational vertical and the yaw axes of the eyes and head (2). In head tilted positions, SVV adjustments are subjected to a systematic bias even in normal subjects, which depends upon the tilt angles. When the head tilt angle is less than 30°, the SVV slightly rotates in the opposite direction of the head tilt, the Müller or E-effect (3). In contrast, the tilt angles of about 30°–60° produce rotation of the SVV in the same direction as the head tilt, the Aubert or A-effect (3).

Deviation of the SVV is one of the most sensitive signs of vestibular disorders that generate imbalance in the graviceptive pathways (4). More than 90% of patients with acute unilateral brainstem infarctions show pathological tilts of the SVV (4). Acute lesions involving the vestibular nerve or the medulla produce ipsiversive tilt of the SVV when measured with head upright (5, 6). Unilateral brainstem lesions affecting the medial longitudinal fasciculus or the interstitial nucleus of Cajal cause contraversive SVV tilt (7, 8). These structures seem to be directly involved in perception of verticality within the multisensory system in which the otolithic graviceptive pathways play a dominant role (7).

A vestibular lesion that leads to SVV tilts in the upright head position may cause perturbations in perception of verticality during head tilts. Perturbed estimation of the SVV during physiological head roll motions impairs balance and causes dizziness whenever patients with vestibular diseases walk and move their head. Only a few studies sought these effects in patients with vestibular disorders. A patient with an acute heminodular stroke showed contralesional tilts of SVV in the primary head and body position, which deviated further in the whole-body roll-tilted position (9). Another study of patients with a hemispheric stroke showed a contraversive SVV tilt in association with visuospatial neglect in the head upright position, and loss of the E-effect when the head was tilted toward the lesion side (10). The authors suggested that the E-effect might be mediated by stretching of the somatosensory receptors of the neck (10). The E-effect observed only during head tilts to the nonparetic side, however, does not support their hypothesis stressing the somatosensory information as an origin of the E-effect. Another study described an enhanced A-effect during lateral body tilt in patients with bilaterally absent vestibular function and also ascribed it to somatosensory inputs (11). Meanwhile, patients with chronic unilateral peripheral vestibular hypofunction showed that adjustment errors of the SVV were pronounced during an ipsilesional tilt, more so in those with ipsilesionally absent ocular vestibular-evoked myogenic potentials (VEMPs) (12). These data support a dominant role of the utricle in perception of verticality during head tilts. Thus, despite the prior studies on the mechanisms of the E- and A-effects (3, 13–15), the origin of these effects remains uncertain.

The SVV has not been assessed systematically during head roll in the body-fixed frame in patients with acute vestibular syndrome, which may reproduce physiologic head roll motion evoked by daily activities. This study for the first time aimed to determine whether erroneous perception of verticality in the upright head position affects the estimation of SVV in head tilted positions during the acute phase of unilateral vestibular disorders. Our assumption was that SVV estimation during head tilts should differ according to the vestibular structures damaged.

## Materials and Methods

### Subjects and Evaluation

This study enrolled 37 patients who presented with acute vestibular symptoms (dizziness/vertigo, unsteadiness, and nausea/vomiting) between March and December 2015. The study population comprised 17 women and 20 men with the mean age at 56.3 ± 14.2 years (range 28–80 years). Patients were excluded if they had impaired attention, or a history of neurologic, vestibular, visual, or spinal problems. Twenty-eight patients had acute unilateral vestibular neuritis (VN). The diagnosis of VN was based on a history of rotatory vertigo lasting several hours to days, a presence of spontaneous contralesional horizontal-torsional nystagmus, positive head impulse tests and caloric paresis on the ipsilesional side, and decreased or no responses of cervical and ocular VEMPs during stimulation of the ipsilesional ear. Eighteen of 28 patients with VN had a utricular involvement when determined by ocular VEMPs, while 23 patients had an involvement of superior division when determined by the video head impulse tests. Nine patients had an acute ischemic stroke restricted to unilateral lateral medulla (see Figure S1 in Supplementary Material). All evaluations were performed during the acute phase with a mean interval of 5.6 days in VN and 5.2 days in lateral medullary infarction (LMI) from the symptom onset. Five of the 28 patients with VN had a follow-up evaluation on average 2 months later during the recovery phase. The demographic characteristics of the subjects are summarized in Table 1.

**Table 1:** Demographic and clinical data of the patients with vestibular diseases.

	Vestibular neuritis	Lateral medullary infarction	Vestibular neuritis, recovery phase
Number of patients	28	9	5
Sex, M/F	14/14	6/3	2/3
Mean age, years (SD)	53.8 (13.5)	64.0 (14.3)	58.6 (7.2)
Mean interval, days	5.6	5.2	61.2
Involved side, R/L	13/15	5/4	5/0

### Experimental Protocol

The SVV was measured in a dark room to eliminate other visual information. Participants sat in front of a screen where a straight luminous rod, 10.0 cm long and 0.5 cm wide, was projected at a distance of 70 cm from the participant’s eyes. Participants were instructed to align the rod vertically, which was presented five times at various angles randomly from the vertical axis. Measurements of the SVV were performed under five conditions in each participant: (I) head upright (PRIMARY), (II) head tilt to the right ear down 30° (RED30), (III) head tilt to the left 30° (LED30), (IV) head tilt to the right 60° (RED60), and (*V*) head tilt to the left 60° (LED60). The tilt angle of the head was measured with a protractor for each condition. An investigator held the participant’s head in position during each of the five trials in a condition. Between conditions, the participant was instructed to hold his/her head upright and to close the eyes for about 30 s. We also adopted the sequence of alternating head tilts in each direction. The SVV tilt in each head position was determined by averaging five adjustments with binocular viewing. For comparison, using the same paradigm, we measured the SVV in 17 healthy controls (6 men and 11 women, mean age 36.7 years, range 28–63 years) who were recruited from laboratory members and colleagues, but were blinded to the goals of the investigation.

### Data Analysis

The SVV tilt was expressed as the deviation from the gravitational vertical (0) measured with a precision of 0.1°. The data were sorted to analyze (1) the SVV tilt in the upright and head tilted positions, and (2) the difference in SVV between the head upright and each head tilted position to estimate a shift in SVV. The mean SVV value was designated as *V*_PRIMARY_, *V*_RED30_, *V*_LED30_, *V*_RED60_, and *V*_LED60_ in each head position. In controls, the SVV was defined positive when the tilt was rightward from the participant’s perspective. In contrast, the SVV (*V* value) was redefined positive when the tilt was toward the lesion side in the patients. The shift in SVV induced by head tilts was obtained by subtracting the SVV in the head upright from the SVV in each head tilted position. In both controls and patients, this calculated shift, designated as “*T*,” was assigned to be negative when the shift directed in the opposite direction of head tilt, indicating the E-effect. Likewise, the positive *T* value indicated that the shift in SVV and the head tilt were in the same direction, i.e., the A-effect.

### Statistics

Testing for the normality of data showed that the distribution of the SVV was Gaussian in both the controls and patients with VN, and data from the controls and patients were presented as the mean and SD. In each upright and tilted head position, the Student’s *t*-test was used for comparisons between controls and patients. In the controls and each group of the patients, the paired *t*-test was used for comparisons between the SVV in the upright and tilted head positions. Statistical analyses were performed using SPSS v.23.0 (IBM Corp., Armonk, NY, USA). Differences were considered significant at *p* values <0.05.

### Standard Protocol Approval, Registrations, and Patient Consents

All experiments followed the tenets of the Declaration of Helsinki. This study was approved by the Institutional Review Board of Seoul National University Bundang Hospital (IRB No. B-1708/412-120). Written informed consents were obtained from the participants.

## Results

### The SVV with the Upright and Tilted Head Positions: Controls

**Figure 1:**
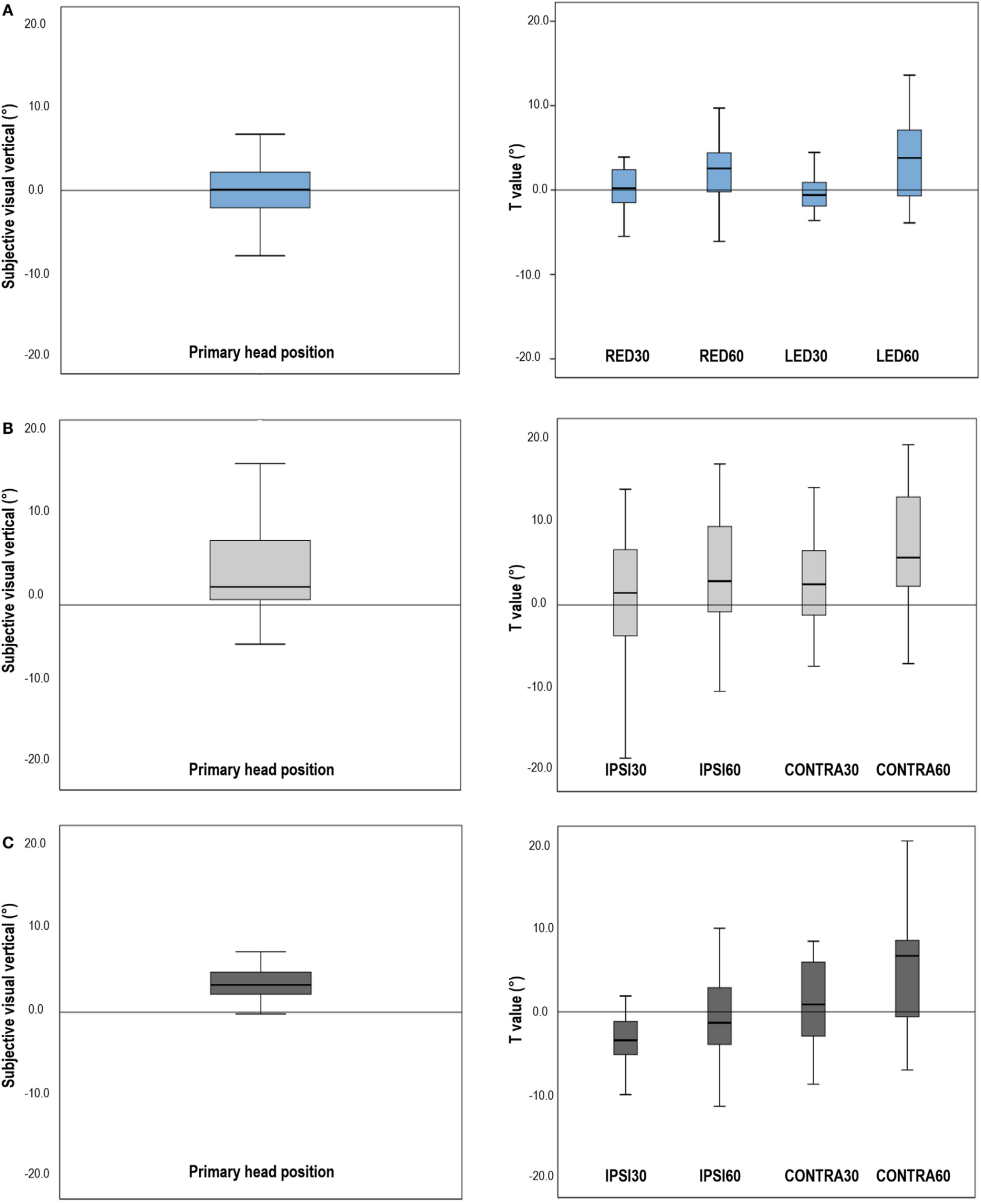

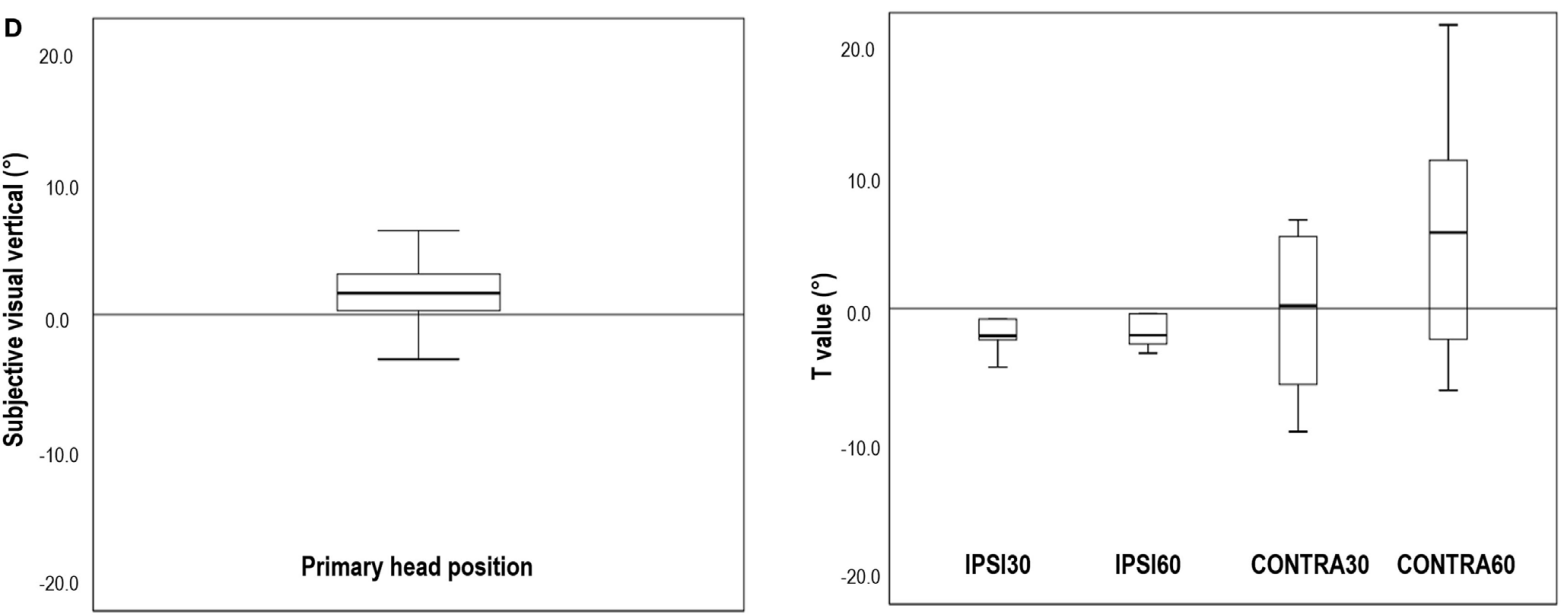
Subjective visual vertical (SVV) in the primary head position and shifts of the SVV induced by head tilts in normal participants and patients. **(A)** In normal subjects, the mean SVV was −0.01 ± 1.20° with the head upright. Head tilts 30° slightly shifted the SVV in the opposite direction of head tilts (E-effect). During head tilts 60°, the shifts of SVV directed toward the head tilted side, resulting in positive *T* values (A-effect). **(B)** In patients with unilateral vestibular neuritis (VN), the SVV in the primary head position was tilted to the lesion side, and head tilts induced shifts of SVV in the direction of head tilts, larger during the contralesional head tilts. **(C)** In patients with lateral medullary infarction (LMI), the SVV in the primary head position was also tilted to the lesion side. Patients with LMI showed negative *T* values during ipsilesional head tilts, indicating the shift of SVV into the contralesional direction, but showed positive *T* values during contralesional head tilts. **(D)** Patients with compensated VN exhibited SVV within the normal range in the upright head position and showed the similar *T* values obtained during head tilts when compared with controls, although the SDs of the *T* values were larger than those of normal subjects. RED30, head tilts 30° with right ear down; RED60, head tilts 60° with right ear down; LED30, head tilts 30° with left ear down; LED60, head tilts 60° with left ear down; IPSI30, head tilt 30° to the ipsilesional side; IPSI60, head tilt 60° to the ipsilesional side; CONTRA30, head tilt 30° to the contralesional side; CONTRA60, head tilt 60° to the contralesional side. Note: In normal controls, a positive value of SVV indicates rightward tilt from the subject’s perspective. In patients, the SVV was defined positive when the tilt is toward the lesion side. The shift in SVV induced by head tilts (*T* value) was obtained by subtracting the SVV in the primary head position from the SVV in each head tilted position. In both normal controls and patients, *T* value was defined to be negative when the shift is in the opposite direction of head tilt. When the shift was in the same direction of head tilt, *T* value was defined to be positive.

In normal controls, tilt of the SVV was −0.01 ± 1.20° with the head upright, which is similar to the normal range reported previously (Figure 1A, left) (16). With the head tilted 30° in each direction, the SVV tended to be tilted in the opposite direction of the head tilts, but remained within the normal range and did not show a statistical difference from the *V*_PRIMARY_ (*V*_RED30_ = −0.05 ± 2.56°, *V*_LED30_ = +0.21 ± 2.84°). In contrast, the SVV tilted in the direction of the head tilts at 60° (*V*_RED60_ = +2.00 ± 4.11°, *V*_LED60_ = −3.41 ± 5.53°). There was no aging effect on the deviation of SVV in the upright and tilted head positions (Pearson correlation test, *p* > 0.05 for all conditions).

Head tilts 30° in controls caused the shifts of SVV in the opposite direction of head tilts (*T*_RED30_ = −0.47 ± 2.93°, *T*_LED30_ = −0.22 ± 2.51°). In head tilts 60°, the shifts of SVV directed toward the side of head tilt (*T*_RED60_ = +2.01 ± 4.32°, *T*_LED60_ = +3.40 ± 5.09°) (Figure 1A, right).

### The SVV in the Upright Head Position: Patients

The SVV in the upright head position was significantly tilted to the lesion side in patients with VN (*V*_PRIMARY_ = +4.21 ± 5.95°, *t*-test *p* = 0.001) (Figure 1B, left) and LMI (+4.13 ± 4.13°, *t*-test *p* = 0.005) (Figure 1C, left). However, patients with compensated VN (+1.58 ± 3.54) showed the mean SVV in the normal range in the upright head position (Figure 1D, left).

### The SVV and Its Shift in the Tilted Head Positions: Patients

Patients with acute VN showed a significant increase in the ipsiversive tilt of SVV during ipsilesional head tilt. In patients with LMI, the shifts in SVV during head tilts was not significantly different from those observed in normal participants (Table 2).

**Table 2:** The subjective visual vertical (SVV) (*V*) and the shifts of SVV during head tilts (*T*).

Normal	*V*_PRIMARY_	*V*_RED30_	*V*_RED60_	*V*_LED30_	*V*_LED60_	*T*_RED30_	*T*_RED60_	*T*_LED30_	*T*_LED60_
	−0.01 (1.20)	−0.05 (2.56)	2.00 (4.11)	0.21 (2.84)	−3.41^†^ (5.53)	−0.47 (2.93)	2.01 (4.32)	−0.22 (2.51)	3.40^†^ (5.09)

**Patients**	***V*_PRIMARY_**	***V*_IPSI30_**	***V*_IPSI60_**	***V*_CONTRA30_**	***V*_CONTRA60_**	***T*_IPSI30_**	***T*_IPSI60_**	***T*_CONTRA30_**	***T*_CONTRA60_**

VN	+4.21* (5.95)	+4.96* (7.27)	+7.79*^†^ (8.03)	+1.29^†^ (4.50)	−2.49^†^ (5.77)	+1.48 (6.90)	+3.58 (7.76)	+2.91* (5.84)	+6.71 (7.00)
LMI	+4.13* (3.26)	+2.16 (6.68)	+3.02 (5.00)	+3.11 (6.59)	−1.30 (9.25)	−2.61 (5.22)	−0.72 (7.04)	+1.02 (5.82)	+5.43 (8.19)
Recovered VN	+1.58 (3.54)	+0.36 (6.04)	+0.72 (4.59)	+2.12 (5.31)	−4.36 (10.83)	−1.22 (2.93)	−0.86 (2.97)	−0.53 (6.89)	+5.94 (10.92)

*RED, head tilts with right ear down position; LED, head tilts with left ear down; IPSI, head tilts to the ipsilesional side; CONTRA, head tilts to the contralesional side; VN, vestibular neuritis; LMI, lateral medullary infarction*.

*The values are presented as the mean (SD)*.

*In normal controls, a positive value of SVV indicates rightward tilt from the subject’s perspective. In patients, the SVV was defined positive when the tilt is toward the lesion side. The shift in SVV induced by head tilts (*T* value) was obtained by subtracting the SVV in the primary head position from the SVV in each head tilted position. In both normal controls and patients, *T* value was defined to be negative when the shift is in the opposite direction of head tilt. When the shift was in the same direction of head tilt, *T* value was defined to be positive*.

*Significantly different from controls.

*^†^Significantly different from the SVV in the primary head position*.

In patients with acute VN, head tilts led to positive *T* values, larger during contralesional head tilts (*T*_CONTRA30_ = +2.91 ± 5.84°, *T*_CONTRA60_ = +6.71 ± 7.00°) rather than during ipsilesional head tilts (*T*_IPSI30_ = +1.48 ± 6.90°, *T*_IPSI60_ = +3.58 ± 7.76°) (Figure 1B, right).

In patients with unilateral LMI, contralesional head tilts also produced positive *T* values (*T*_CONTRA30_ = +1.02 ± 5.82°, *T*_CONTRA60_ = +5.43 ± 8.19°) whereas ipsilesional head tilts produced negative values (*T*_IPSI30_ = −2.61 ± 5.22°, *T*_IPSI60_ = −0.72 ± 7.04°) (Figure 1C, right). In patients with LMI, the shift of SVV induced by head tilt did not show a statistical difference from that of control.

Patients with compensated VN showed the mean SVV within the normal range during both head upright and tilted positions. Compared to controls, patients with VN during the recovery phase did not show statistical differences in the *T* values, although the SDs were larger than those of control (Figure 1D, right).

## Discussion

This study determined the influence of head tilts on perception of SVV in acute vestibular disorders. In healthy subjects, a bidirectional pattern of bias was found when estimating the SVV during head tilts, but within a narrow range. Head tilts 30° caused the shift of SVV in the opposite direction of head tilt, and head tilts 60° caused the shift of SVV in the same direction of head tilt. In patients with acute disorder involving the peripheral or central vestibular structures, average estimation errors of SVV induced by head tilts were larger than those in controls, and often showed unidirectional shifts (Table 3).

**Table 3:** Summary of the shifts of subjective visual vertical during head tilts.

	E-effect	A-effect
Normal control	In small head tilts	In large head tilts
Vestibular neuritis (VN)	–	Dominant in contralesional head tilts
Lateral medullary infarction	In ipsilesional head tilts	In contralesional head tilts
VN, during the recovery phase	In small head tilts	In large head tilts

The prior perception of tilted verticality in the primary head position may have affected the following SVV estimation during head roll tilts, but that was not an only determinant of SVV shifts induced by head tilts. Estimation error of SVV was asymmetric during ipsilesional and contralesional head tilts in our patients, rejecting the hypothesis that a constant offset is added to physiological deviation in SVV estimation when roll-tilted. The increased estimation errors during head tilts to one side in our patients may be related to increased weighting of prior knowledge on tilted verticality when the vestibular inputs became less reliable due to the injury. To explain the unequal shifts of SVV during head tilts in either direction, however, an asymmetric increase in the weight of prior perception would be required. In addition, the mechanical properties of otolithic organs may provide an explanation.

**Figure 2:**
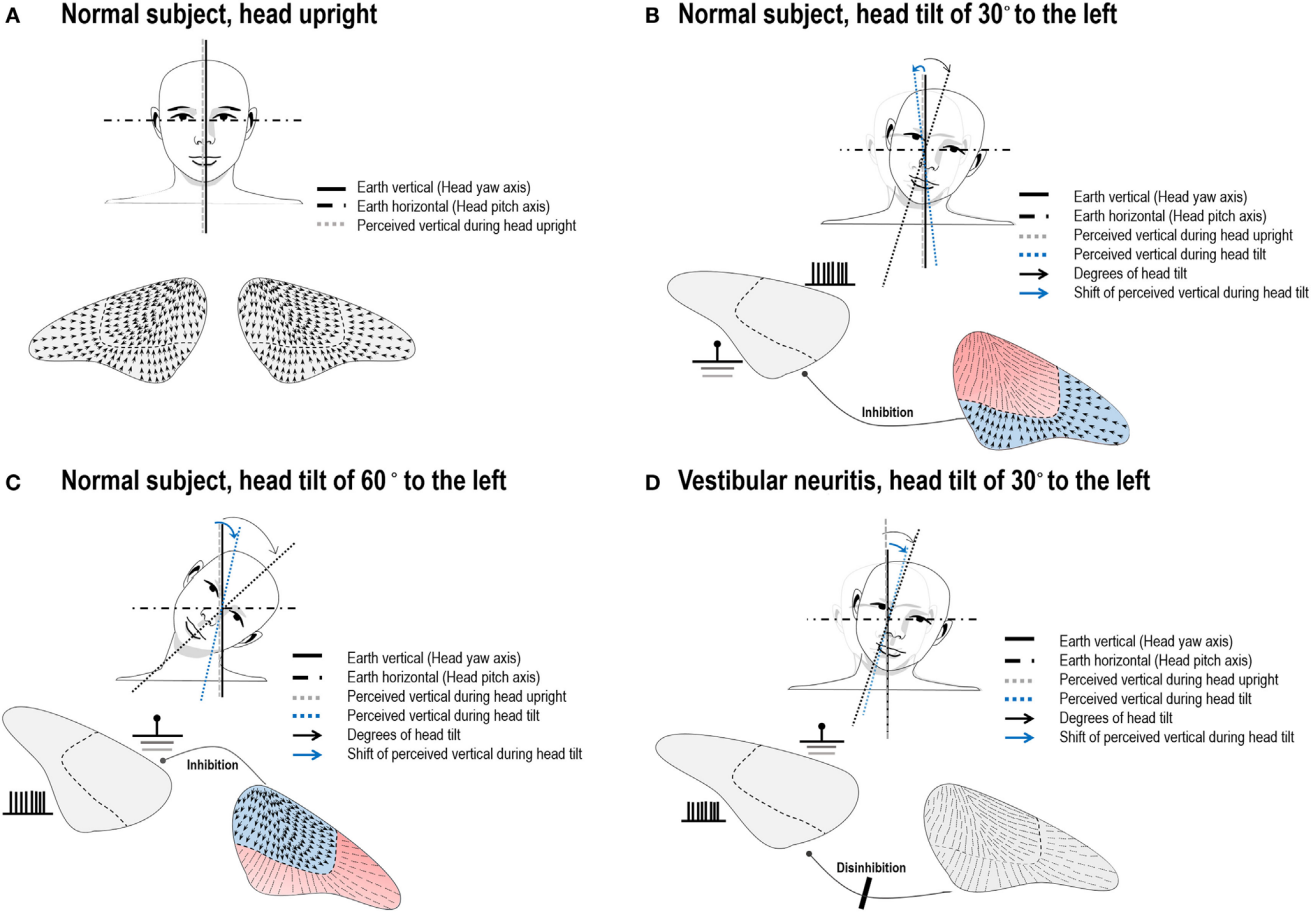
Illustration of arrangement of the utricular hair cells and hypothetic neuronal activities during head tilts in normal subjects and in a patient with left vestibular neuritis (VN). **(A)** The hair cells of an opposite polarization are oriented toward the striola of the utricular macula. In normal subjects with the head upright, the subjective visual vertical (SVV) is aligned with the gravitational vertical and the yaw axes of the eyes and head. **(B)** Head tilts of small angles would activate the lateral portion of the utricle on the tilted head side. This would result in overall deflections of the hair cells in the opposite direction of a head roll tilt, and hence lead to a small contraversive shift of the SVV (E-effect). **(C)** We hypothesize that larger head tilts would more strongly activate the medial portion of the utricle on the head tilted side, while the neuronal activities on the lateral portion in the ear on the side of head tilt and on the medial portion in the other ear are inhibited. This would result in the net neuronal activities directing toward the side of a head tilt, resulting in a shift of the SVV in the direction of head tilt (A-effect). **(D)** If a patient with left VN slightly tilts his/her head to the ipsilesional side, afferent signals from the lateral portion of the utricle cannot be generated in the left ear and commissural inhibition of the activities from the corresponding portion of the utricle in the right ear does not occur. Thus, the disinhibited neuronal activities from the intact ear would deviate the SVV in the direction of head tilt, resulting in A-effect. Blue colored portions indicate activation of the hair cells while red colored portions represent inhibited neuronal activities.

The SVV primarily reflects asymmetry of utricular inputs between the sides of the vestibular system (12). The utricle responds to roll tilts and side-to-side translation of the head (17). The hair cells of an opposite polarization are aligned on either side of the striola that divides the maculae into two parts. In the utricular macula, the hair cells are oriented toward the striola, with a 3:1 preponderance of the units with ipsilaterally directed vectors (Figure 2A) (18). The medial and lateral portions of the utricle would respond differently according to the vectors generated by different degrees of head tilt. When the head is tilted in the direction of a unit of the hair cell’s axis of polarity, that cell depolarizes and excites the afferent vestibular fiber. During linear acceleration of the head, some hair cells are depolarized while others on the opposite side of the striola are hyperpolarized (cross-striolar inhibition) (19). This mechanism enables that each otolith organ is independently capable of producing an electrical asymmetry necessary for detecting motion (19, 20). Due to their mechanical properties, the firing rates of the utricular hair cells may depend proportionally on membrane displacements that vary upon the angles of head tilts. That is, head tilts below 30° would mainly excite the lateral portion of the utricle (21) and induce a small deflection of the hair cells in the opposite direction of head movements, which can contribute to the E-effect (Figure 2B). At the same time, secondary afferents from the lateral portion of the utricle in the opposite ear would be inhibited *via* the commissural projections (19, 21). Although there have been no experimental data on the excitatory patterns of the hair cells in the medial and lateral portions of the utricle during head tilts more than 60°, the medial portion of the utricle would be more excited by the changes in the vertical vectors produced by larger head tilts. Thus, we hypothesize that head tilts more than 60° may lead to greater deflection of hair cells in the direction of head tilt for the ipsilateral ear and that this excitation would lead to cross-striolar inhibition of the lateral portion of the ipsilateral utricle, as well as inhibition of the secondary afferents from the medial portion of the contralateral utricle *via* commissural projections (Figure 2C). This pattern of activation and inhibition within and between ears seems to explain a narrow range of SVV tilts in normal subjects even during larger head tilts. Although our hypothesis explains the mechanisms of the A- and E-effects based on the interactive connections of the utricles and the otolithic pathways, proprioception may also contribute for generation of those effects (10, 11).

Patients with VN showed the A-effect irrespective of the directions of head tilts. Given the loss of the E-effect in patients with unilateral VN, this effect seems to require normal function of the peripheral otolithic organs on both sides. If a patient with unilateral VN slightly tilts his/her head to the ipsilesional side, afferent signals from the lateral portion of the utricle cannot be generated in the ipsilesional ear, while simultaneous inhibition of the secondary afferents from corresponding portion of the utricle in the intact ear does not occur due to absence of the signals from the ipsilesional ear that pass through the commissural projections (Figure 2D). The net effect would be the neuronal activities from the intact ear mainly directing toward the direction of head tilt. This A-effect would be greater if the patient with VN tilts his/her head toward the intact ear since the firing due to direct stimulation are stronger than those due to disinhibition (22). Our patients with acute VN indeed showed pronounced A-effect during contralesional head tilts. Meanwhile, the SVV testing in patients with VN in the recovery phase indicates that perturbations of perceived verticality during head roll improve in parallel with the recovery after peripheral vestibular injury.

On the contrary, patients with unilateral LMI showed normal patterns of SVV shift during head roll tilt, regardless of its amplitude and direction, although they had underlying biased perception of gravity during the head upright position. Given that the principal brainstem areas of termination of the utricular afferents are the medial and superior vestibular nuclei (23), a focal injury of the central otolithic pathways at the caudal level of the lateral medulla seemed to preserve the capability of estimating gravity direction during the changes in head roll positions.

In patients with unilateral lesions involving the peripheral or central vestibular structures, the SVV shifts induced by head tilts were larger than those in normal controls and often showed a unidirectional pattern irrespective of tilting direction or angle. Given the absence of the E-effect in acute VN, the peripheral otolithic inputs appear to be essential for estimation of earth vertical during small head tilts.

## Ethics Statement

All experiments followed the tenets of the Declaration of Helsinki. This study was approved by the Institutional Review Board of Seoul National University Bundang Hospital (IRB No. B-1708/412-120). Written informed consents were obtained from the participants.

## Author Contributions

S-HK planned and conducted the experiments, analyzed and interpreted the data, and wrote the manuscript; J-SK conducted the design of the study, interpreted the data, and revised the manuscript.

## Conflict of Interest Statement

The authors declare that the research was conducted in the absence of any commercial or financial relationships that could be construed as a potential conflict of interest.

## Supplementary Material

Figure S1 Brain MRI of nine patients with unilateral lateral medullary infarction.
