# Supplementary figures and images for: Effects of Head Position on Perception of Gravity in Vestibular Neuritis and Lateral Medullary Infarction
Source: Front Neurol. 2018 Feb 12;9:60. doi: 10.3389/fneur.2018.00060 (PMC5816270; doi:10.3389/fneur.2018.00060)

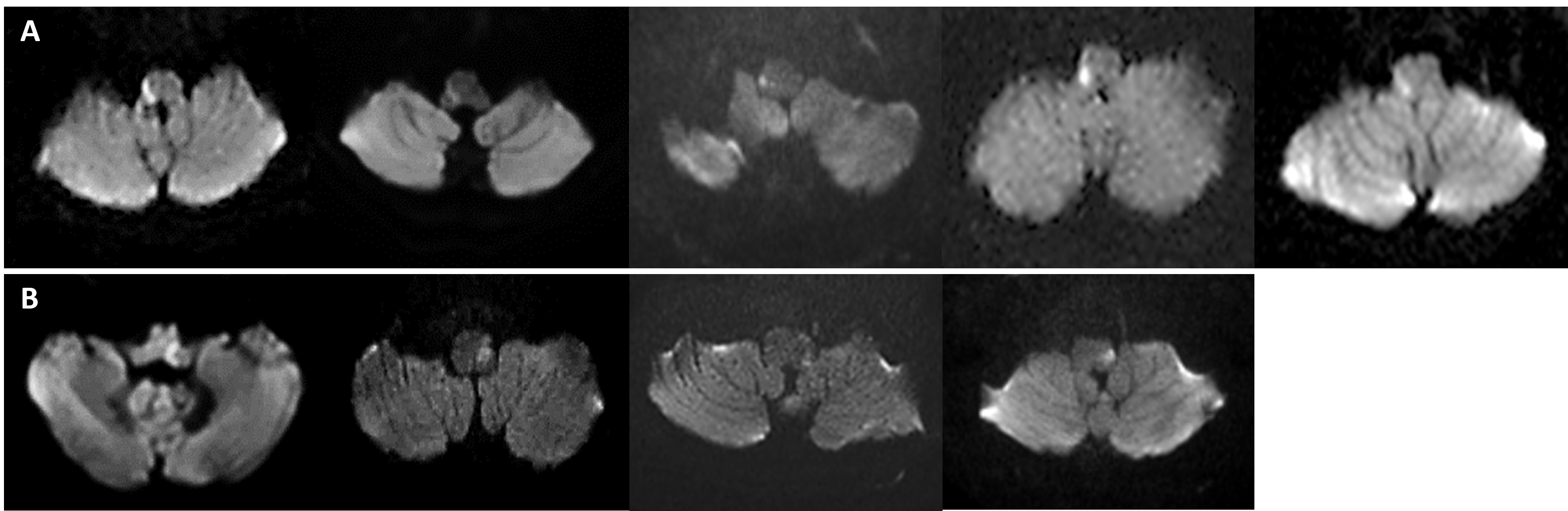

Supplement: Figure S1 — Brain MRI of nine patients with unilateral lateral medullary infarction. [file image_1.tif]
